# The relationship between jumping to conclusions and social cognition in first-episode psychosis

**DOI:** 10.1038/s41537-022-00221-3

**Published:** 2022-04-20

**Authors:** Luciana Díaz-Cutraro, Raquel López-Carrilero, Helena García-Mieres, Marta Ferrer-Quintero, Marina Verdaguer-Rodriguez, Ana Barajas, Eva Grasa, Esther Pousa, Ester Lorente, María Luisa Barrigón, Isabel Ruiz-Delgado, Fermín González-Higueras, Jordi Cid, Laia Mas-Expósito, Iluminada Corripio, Irene Birulés, Trinidad Pélaez, Ana Luengo, Meritxell Beltran, Pedro Torres-Hernández, Carolina Palma-Sevillano, Steffen Moritz, Philippa Garety, Spanish Metacognition Group, Susana Ochoa

**Affiliations:** 1 Etiopatogènia i tractament dels trastorns mentals greus (MERITT), Institut de Recerca Sant Joan de Déu, Esplugues de Llobregat, Barcelona, Spain; 2 Parc Sanitari Sant Joan de Déu, Sant Boi de Llobregat, Barcelona, Spain; 3 Psychology Department, FPCEE Blanquerna, Universitat Ramon Llull, Barcelona, Spain; 4 Investigación Biomédica en Red de Salud Mental (CIBERSAM), Madrid, Spain; 5 Social and Quantitative Psychology Department, University of Barcelona, Barcelona, Spain; 6 Centre d’Higiene Mental Les Corts, Barcelona, Spain; 7 Departament de Psicologia Clínica i de la Salut, Facultat de Psicologia, Universitat Autònoma de Barcelona, Barcelona, Spain; 8 ‘Serra Húnter fellow’, Barcelona, Spain; 9 Department of Psychiatry, Hospital de la Santa Creu i Sant Pau, Institut d’Investigació Biomèdica-Sant Pau (IIB-Sant Pau), Barcelona, Spain; 10 Psychiatry Service, Hospital Clínico Universitario de Valencia, Valencia, Spain; 11 Servicio Andaluz de Salud, Area de Gestión Sanitaria Sur Granada, Motril, Spain; 12 Department of Psychiatry, IIS-Fundación Jiménez Díaz Hospital, Madrid, Spain; 13 Universidad Autónoma de Madrid, Madrid, Spain; 14 Unidad de Salud Mental Comunitaria Malaga Norte. UGC Salud Mental Carlos Haya, Servicio Andaluz de Salud, Málaga, Spain; 15 Comunidad Terapéutica Jaén Servicio Andaluz de Salud, Jaén, Spain; 16 Mental Health & Addiction Research Group, IdiBGi, Institut d’Assistencia Sanitària, Girona, Spain; 17 Hospital de Mataró, Consorci Sanitari del Maresme, Barcelona, Spain; 18 Department of Psychiatry and Psychotherapy, University Medical Center Hamburg, Hamburg, Germany; 19 Institute of Psychiatry, Psychology & Neuroscience, King’s College London, London, UK

**Keywords:** Psychosis, Diseases

## Abstract

Jumping to conclusions (JTC) and impaired social cognition (SC) affect the decoding, processing, and use of social information by people with psychosis. However, the relationship between them had not been deeply explored within psychosis in general, and in first-episode psychosis (FEP) in particular. Our aim was to study the relationship between JTC and SC in a sample with FEP. We conducted a cross-sectional study with 121 patients with FEP, with measures to assess JTC (easy, hard, and salient probability tasks) and SC (emotional recognition, attributional style, and theory of mind). We performed Student’s t-test and logistic regression in order to analyse these associations.We found a statistically significant and consistent relationship of small-moderate effect size between JTC (all three tasks) and impaired emotional recognition. Also, our results suggest a relationship between JTC and internal attributions for negative events. Relationships between JTC and theory of mind were not found. These results highlight the importance of psychological treatments oriented to work on a hasty reasoning style and on improving processing of social information linked to emotional recognition and single-cause attributions.

## Introduction

‘Jumping to Conclusions’ (JTC) is a reasoning bias that consists of a tendency to make hasty decisions and judgments about an event without having enough information to obtain correct and functional ideas or conclusions, resulting in the lack of consideration of more flexible alternative explanations and consequently, incorrect reasoning^1–3^. People with psychosis show a pronounced hasty decision-making style^4–7^ in different stages of the illness (including those with high risk and patients with symptomatic remission), suggesting that it is a stable pattern^8^ and a cognitive trait marker for psychosis^5^. Likewise, a higher prevalence of JTC is related to delusional symptomatology^9,10^, in people with psychosis and in the general population^11^. In this sense, it is hypothesised to be involved in the formation and maintenance of delusional beliefs^4,5,12,13^. Moreover, a greater predisposition to JTC predicts a worse prognosis for delusions^4,14–16^. Likewise, we know that JTC in people with first episode psychosis (FEP) is associated with more implausible delusional subtypes, such as control delusions and magical thinking^17^. Additionally, JTC has been associated with lower neuropsychological functioning in psychosis^18–21^.

In the last decade the interest in the study of social cognition (SC) in FEP has been growing^22^. SC is a series of cognitive processes related to the management of social information, coding, storage, recovery, and its application to social situations^23^. SC is closely related to social and general functioning in people with psychosis, even to a greater extent than neurocognition, hence providing independent and more ecological predictions of functioning^24–27^. It is a multifaceted construct, and through the SCOPE project^28^ a consensus has been generated postulating that it is composed of four subdomains: Emotional Processing (EP), Theory of Mind (ToM), Attributional Style (AS) and Social Perception (SP)^28–30^. EP consists of identifying and correctly using emotions; this domain also involves Emotion Recognition (ER). ToM evaluates the ability to infer mental states in other people; AS refers to the ability to explain causes and make sense of social interactions and situations, and SP consists of interpreting social signals. A detailed and comprehensive review by Healey et al. (2016) showed that people with FEP present deficits in ER and ToM^22^.

The relationship between JTC and SC has not been deeply examined. Tripoli et al.^31,32^ reported that people with FEP who have JTC tend to have low EP; in contrast, there was no association between JTC and AS^31^. Regarding the relationship between JTC and ToM, Takeda^33^ and Woodward et al.^34^ did not find associations. These studies analyse the relationship with each domain separately in populations with long-term schizophrenia and not with people first-episode psychosis^33,35^. On the other hand, Grossman and Bowie^27^, assessed JTC and SP in people with FEP, and found that overconfidence in making social judgments resulted in insufficient or erroneous conclusions when processing social information. Therefore, further studies evaluating the relationship between JTC and CS in a global and exploratory manner are needed. Also, studies exploring this relationship at different stages of psychosis are needed to know whether the possible relationships are present and in the same way along the psychosis continuum. Understanding the relationship between JTC and SC could also be of great help for the development and personalisation of gold standard interventions that work together with reasoning biases in the social decision-making in patients with psychosis.

Our objective was to explore the relationship between JTC and three domains of CS. Furthermore, we controlled for possible confounders (estimated premorbid IQ, positive and depressive symptoms), and we explored the most relevant variables of social cognition that explain the presence of JTC.

## Results

### Socio-demographic and clinical characteristics

Table 1 shows the sociodemographic and clinical characteristics of the sample. The percentage of women is 30.30%, and that of men is 69.7%. The mean age is 27.59 (SD = 7.39), the months of illness are 25.57 (SD = 7.22), and the number of previous suicide attempts is 1.18 (SD = 0.39).

**Table 1:** Description of sociodemographic and clinical variables of the sample.

	*N*	%
Men	85	69.7
Women	37	30.3
	**Mean**	**SD**
Age	27.59	7.39
Month of evolution of illness	25.57	7.22
Number of previous suicide attempts	1.18	0.39
Number of hospitalisations	1.24	1.40
Chlorpromazine equivalent dose	494.77	627.12
PANSS positive	12.21	4.14
PANSS negative	14.64	6.00
PANSS general	27.43	6.78
PANSS total	54.33	14.31
PDI total experiences	6.04	4.68
PDI distress	14.88	16.08
PDI preoccupation	15.08	16.76
PDI level of conviction	18.08	17.70
GAF Total	61.26	12.71

*PANSS* Positive and Negative Syndrome Scale, *PDI* Peters Delusions Inventory, *GAF* Global assessment of functioning. The Global Assessment of Functioning (GAF) scale measures the extent to which a person’s symptoms affect functioning in different spheres of daily life, with a scale from 0 to 100^18,76^.

### Participants who did JTC on Beads Task

Table 2 shows the percentages of participants who jump to conclusions and those who do not, in each of the beads tasks. A total of 29.8% (*n* = 36) of the individuals jumped to conclusions in Task 1, 14.0% (*n* = 17) in Task 2, and 15.7% (*n* = 19) in Task 3.

**Table 2:** Percentages of participants who jump to conclusions and those who do not in each bead task.

Task 1 - 85%:15%				Task 2 - 60%:40%				Task 3 - Affective 60%:40%			
JTC		Non-JTC		JTC		Non-JTC		JTC		Non-JTC	
N	%	N	%	N	%	N	%	N	%	N	%
36	29.8	85	70.2	17	14	104	86	19	15.7	102	84.3

### The relationship between JTC and SC

Table 3 shows the relationship between those who perform JTC and who do not and SC variables. Firstly, people who jumped to conclusions in all three tasks scored lower in ER than those who did not. The easiest JTC task (85:15) showed statistically significant differences on SC measures with small effect sizes, while the 60:40 task and the salient task showed medium effect sizes. Regarding ToM, we have found no relationships with JTC. Considering AS, we found mixed findings. Persons who JTC make more internalized attributions for negative events, in task 1 and 3, than people who do not JTC. Likewise, we found a relationship between JTC and low externalized bias in task 1, and with situational attribution of negative events in task 3.

**Table 3:** Comparison of people who jumped to conclusions or not, regarding attributional style, emotional recognition, and ToM, using Student’s t-tests.

			JTC 85:15			JTC 60:40			JTC 60:40 salient task		
			Mean	SD	*P*-value (Effect size)	Mean	SD	*P*-value (Effect size)	Mean	SD	*P*-value (Effect size)
ER	Emotional recognition test	No JTC	17.81	1.58	0.042 (0.388)*	17.74	1.67	0.031 (0.539)**	17.82	1.54	0.017 (0.721)**
		JTC	17.11	2.00		16.76	1.95		16.42	2.27	
ToM	Hinting task	No JTC	4.81	1.09	0.308 (0.202)	4.76	1.10	0.703 (0.092)	4.79	1.06	0.255 (0.258)
		JTC	4.58	1.18		4.65	1.27		4.47	1.39	
Attributional style	Internal attribution of positive event	No JTC	7.14	3.40	0.745 (0.064)	7.05	3.43	0.202 (0.331)	7.12	3.30	0.522 (0.152)
		JTC	7.37	3.74		8.25	3.80		7.72	4.51	
	Internal attribution of negative event	No JTC	5.96	3.65	0.034 (0.425)*	6.21	3.62	0.103 (0.401)	6.12	3.55	0.029 (0.509)**
		JTC	7.57	3.93		7.88	4.63		8.22	4.63	
	Personal attribution of positive event	No JTC	4.94	3.52	0.225 (0.255)	4.67	3.42	0.822 (0.063)	4.71	3.41	0.907 (0.029)
		JTC	4.11	2.96		4.88	3.16		4.61	3.26	
	Personal attribution of negative event	No JTC	6.77	3.66	0.125 (0.313)	6.52	3.59	0.547 (0.145)	6.50	3.50	0.672 (0.100)
		JTC	5.66	3.41		5.94	3.84		6.11	4.25	
	Situational attribution of positive event	No JTC	3.92	3.33	0.516 (0.1126)	4.27	3.51	0.077 (0.514)	4.16	3.45	0.423 (0.204)
		JTC	4.37	3.79		2.63	2.83		3.44	3.58	
	Situational attribution of negative event	No JTC	3.23	3.32	0.389 (0.176)	3.21	3.35	0.191 (0.386)	3.33	3.38	0.004 (0.636)**
		JTC	2.66	3.14		2.06	2.54		1.56	2.01	
	Externalised bias	No JTC	1.18	4.06	0.042 (0.388)*	0.83	3.76	0.658 (0.112)	1.00	3.82	0.124 (0.404)
		JTC	−0.20	2.95		0.38	4.22		−0.50	3.59	
	Personalised bias	No JTC	1.27	0.68	0.929 (0.028)	1.23	0.67	0.073 (0.470)	1.24	0.66	0.184 (0.315)
		JTC	1.29	0.71		1.56	0.73		1.47	0.79	

*Low effect size.

**Medium to large effect sizes.

Bold values identify statistical significance.

### SC and confounding variables explaining JTC

In order to examine the effect of depression (BDI), positive symptoms (PANSS) and IQ (WAIS) as confounders, a logistic regression analysis was performed including these variables to the previous analysis. Table 4 shows a logistic regression analysis where only statistically significant variables were included. JTC in Task 1 was explained by ER and AS (Internal attributions for negative events). In the Task 2, JTC was explained by ER. Finally, in the case of Task 3, JTC was explained by ER and IQ.

**Table 4:** Logistic regression model of social cognition and clinical variables associated with jumping to conclusions.

		Task 1 - 85%:15%			Task 2 - 60%:40%			Task 3 - Salient 60%:40%		
		*B*	SE	*p*-value	*B*	SE	*p*-value	*B*	SE	*p*- value
Emotional recognition	Emotional recognition test	−0.329	0.134	**0.014**	−0.472	0.171	0.006	−0.691	0.228	**0.002**
Attributional style	Internal attribution for negative events	0.13	0.061	**0.031**	-	-	-	-	-	0.058
	Situational attribution for negative events	-	-	-	-	-	-	-	-	0.167
	Externalizing bias	-	-	0.539	-	-	-	-	-	-
Clinical and IQ variables	Depressive symptoms	-	-	0.096	-	-	0.513	-	-	0.358
	Positive symptoms	-	-	0.864	-	-	0.899	-	-	0.171
	Estimated premorbid IQ	-	-	0.107	-	-	0.083	−0.061	0.022	**0.005**
Model R2 Nagelkerke		0.146			0.131			0.426		

Bold values identify statistical significance.

## Discussion

Our aim was to examine the relationship between JTC and different domains of SC in people with FEP, and to explore what of these components of social cognition explain the presence of JTC. We found several results that merit discussion in this study. First, we found an association between JTC and ER, and between JTC and some aspects of AS (almost with internal attribution of negative events), but there was no evidence of a relationship between JTC and ToM. The effect sizes of the relationships between JTC and ER were greater in the most discriminating tasks (tasks of the proportion 60:40 with beads and salient variant); additionally, ER explains the variance of JTC in the three tasks.

Regarding emotion recognition, we found that patients who jump to conclusions have more difficulty recognising emotions than those that do not present the JTC bias, even after controlling for mood and positive symptoms. These results highlight the need for improving reasoning judgments that are involved in ER in people with FEP. The results are consistent with the previous findings by Tripoli et al.^31,32^ where it was observed that patients with FEP who displayed JTC recognised worse emotions globally compared with controls, which is especially important because few psychological treatments intervene in both constructs^36^. In this line, psychological interventions such as metacognitive training for psychosis (MCT) would be recommended, because it works with specific modules about ER and the importance of making judgments based on the search of sufficient evidence to do that^37,38^. The MCT invites participants to “sow the seed of doubt” in the inflexible thinking present in psychosis^39^. Furthermore, it could be suggested the indication of this intervention, because the improvements at the end of the MCT are maintained in the follow-up^40–42^. This is a “sleeper” effect of MCT, which could have an important effect in the future of patients with early stages of the psychosis and could prevent deficits in social information processing in more advanced stages of psychosis^41^. In conclusion, for patients with FEP who do JTC, it seems relevant to us to recommend early psychological treatments focused on modifying reasoning biases and improving the recognition of facial emotions.

Considering ToM, we did not find a relationship between JTC and ToM. That is, those people who have the JTC bias do not necessarily interpret the mental states of others more poorly than people who do not JTC. Our findings are consistent with previous findings by Woodward et al.^32^. Using factor analysis, these authors found that JTC loaded in a different neurocognitive factor than ToM. Likewise, Takeda et al.^31^ did not find a relationship between JTC and ToM. However, it should be noted that they found a relationship between decision confidence and ToM. Patients who tended to make interpretations with a high level of confidence failed to make appropriate interpretations of the mental states of the others. There are methodological differences between our study and theirs, as we used different measures for SC, and we did not measure confidence in the decision. Furthermore, their sample consisted in participants with long-term schizophrenia. Further studies should assess the relationship between ToM and JTC considering them as independent mechanisms.

Regarding Attributional style, our results suggest that people who do JTC easily (in task 1) and/or in decisions more influenced by social content (task 3) would tend to make unidirectional attributions either to themselves or to situations in a negative way, even after controlling for mood, positive symptoms, and IQ. In order to discuss the findings with previous literature, as we have not found studies that previously analysed this relationship in FEP, we consider the results of other studies on psychotic spectrum disorders in more advanced stages of the disorder, or of studies based on AS only. Based on what we found, we infer those patients with FEP who make hasty decisions tend to assign the cause of negative events to themselves or to situations. In contrast to our findings, Merrin et al.^43^ observed that JTC, in patients with persecutory delusions, is related to the greater presence of an external/personalising bias. On the other hand, Moritz et al. found no relationship between JTC and AS^35,44,45^, although it should be taken into account that Moritz et al.^35,44^ measure AS in a different way than we do, and this may influence the results. However, they found a contrast with previous findings highlighting that patients with paranoia tend to blame others for negative events^9^, suggesting that patients with schizophrenia displayed a ‘depressive realism’ (no further credit attributed to positive events than they blame themselves for negative events). This could be explained by the theory of Trower and Chadwick^46^ of the ‘bad me/poor me’ of paranoia^47^ where the ‘poor me’ is characterised by seeing themselves as an innocent victim while condemning others for the persecution. In this vein, the attributional judgments of the patients could be labile, as they are able to go from being personalising/external to blaming themselves after experiences of failure^48^. Several psychological interventions address work with JTC and AS, such us the MCT and SlowMo therapy^49^. This last intervention is an innovated blended digital intervention targeting reasoning to help reduce paranoia and has been found to be a highly engaging treatment option^49,50^. This treatment merges the benefits and the attraction of the new technologies with a focus on key mechanisms, such as JTC and AS, that play a central role in psychotic symptoms.

Taking into account the confounders that we considered, neither depressive symptoms nor negative symptoms explained the presence of JTC, although IQ in JTC task 3 did. This finding is related to previous studies such as Tripoli et al.^21^ and Gonzalez et al.^19^ However, Ochoa et al.^18^ and Garety et al.^43^ did not find a relationship between JTC and IQ, although it was found with other neuropsychological variables. Tripoli et al.^21^ found that JTC may be associated with psychosis through IQ. However, only JTC would be an indicator of good/bad prognosis in professional status (Andreou et al. 2014), in delusional symptomatology^4^, in hospitalizations and compulsory admissions^3^. In our results, IQ only explained the presence of JTC in task 3 (one of the two tasks considered difficult) and we suggest that further studies should be conducted that consider neuropsychological variables as mediating variables in the relationship between JTC and CS to study the role of IQ in greater depth in this population.

Some limitations should be considered in the methods of this study. Regarding measurement in the JTC assessment, we did not measure confidence in decisions. Considering the measurement of SC, the task used to evaluate EP assesses a basic level of ER, making it necessary to include more complex measures in future studies. Depressive symptoms did not modify the results of JTC and social cognition, however further studies should consider using a more specific scale for the assessment of depressive symptoms in psychosis such as Calgary Depression Scale. Regarding the sample, another limitation is that we did not have healthy controls as a comparison group. It should be considered, the low percentage of people that JTC in comparison with other studies which may have led to a floor effect; one possible explanation could be related with the early stage of illness of the sample (noting that some reports have found lower levels of JTC in the early stages than in chronic stages)^18^. As an inclusion criterion was that the subject had positive symptoms, the results are generalizable only to the population with more psychotic symptoms or those who are more similar to the population that we can attend in specific services for the rehabilitation of people with FEP.

Future studies should analyse the possible mediating effect between JTC and SC of more comprehensive neuropsychological variables (memory, attention, perception, executive functions)^19,21^ and insight^51,52^. Studies that include these domains as mediators can help to deepen the understanding of the nature of the relationship between JTC and the SC. For this part, longitudinal and comparative studies on JTC and SC in different stages of psychosis could be beneficial for personalised treatments according to the stage of illness. Similarly, studies involving more complex social cognition measurements may shed light on actual results.

As a conclusion, our results suggest that patients who are quick to draw conclusions have impairment in processing social information, which involves poorer ER, and an AS based on unilateral attributions oriented to a negative internalisation for the causes of events. These findings may suggest common psychopathological constructs between JTC, ER, and one-sided self-negative attributions that can be worked on in psychological interventions that include them in their therapeutic objectives. Given that our sample in this study represents a population with ongoing acute symptoms, this finding is of special importance for those health services designed for interventions in the early phases of psychosis, such as rehabilitation services, first episode programmes for psychotic patients, or inpatient services. Therefore, psychological interventions that address hasty reasoning style in patients with psychosis should consider exercises and clinical examples that help them improve processing social information and considering multiple causes for social and non-social events, and a correct recognition of emotions in others.

## Methods

### Study design

A cross-sectional study was performed based on baseline data from a large multicentre clinical trial^40^. The study was recorded in Clinical Trials (Identifier: NCT02340559).

### Sample, inclusion, and exclusion criteria

The sample was composed of 121 patients with a FEP^53^ recruited at one of the nine participating mental health centres: Servicio Andaluz of Jaén, Málaga and Motril (Granada), Hospital de la Santa Creu i Sant Pau (Barcelona), Hospital Clínico Universitario de Valencia, Centro de Higiene Mental de les Corts (Barcelona), Salut Mental Parc Taulí (Sabadell), Institut d´Assistència Sanitària Girona, and the coordinating centre Parc Sanitari Sant Joan de Déu (Sant Boi).

Regarding the proposal of Breitborde et al.^53^ inclusion criteria were: (1) a diagnosis of schizophrenia, psychotic disorder not otherwise specified, delusional disorder, schizoaffective disorder, brief psychotic disorder, or schizophreniform disorder (according to DSM-IV-TR); (2) < 5 years from the onset of symptoms; (3) PANSS scores in delusions, grandiosity, or suspiciousness of ≥ 4 during the previous year; and (4) age between 17 and 45 years. Exclusion criteria were: (1) a traumatic brain injury, dementia, or intellectual disability (premorbid IQ ≤ 70; (2) substance dependence; and (3) PANSS scores in hostile and uncooperativeness of ≥ 5 and in suspiciousness ≥ 6.

### Assessment

Considering the assessment, we included a sociodemographic questionnaire which collects relevant information regarding the description of the samples. We also included different questionnaires regarding JTC, SC, and clinical measures.

### Assessment of jumping to conclusions bias

JTC was assessed with the computer version of the beads task^54,55^ in which the participants have to make the decision from which jar the extracted beads come from. All tasks have an introductory part, in which the two jars (in the case of the 85:15 and 60:40 beads task)/studies (in the case of the salient task) are presented, and an instructional part, in which it is explained to the participant what he/she must do. The first part is similar in the explanations of the three tasks, here is a quote from one of them, in this case from the salient version: “Imagine that we have conducted two studies on a person very similar to you. The study with majority negative gave 60 negative comments and 40 positive comments. The study with majority positive gave 60 positive comments and 40 negative comments. The positive comments are words like kind, decent, warm. Negative comments are words like vain, cold, greedy”. The instructions according to the task were, “One of the jars (in the case of the beads) or studies (in the case of the outgoing task) has been randomly chosen. Comments will be drawn from the selected jar/study and shown to you. The comments will always come from the same jar/study and will be substituted so that the proportions remain the same. You decide which jar/study the comments come from. You can see as many comments as you want before deciding. After you are shown a bead/comment, you can ask for another bead/comment or you can tell me that you know which jar/study was chosen and whether it is the study with the most negatives or the study with the most positives (or the jar with the most black/purple or orange/green beads). Remember that you can look at as many comments as you want before deciding which study the comments are from. Decide only when, you are sure. Now you will see the first bead/comment.” Next, for each bead/comment the participant asks to see, the following question is offered, “Are you ready to decide or do you want to see more beads?” In task 1, one jar is presented with a ratio of 85% black beads to 15% orange beads while a second jar has the inverse proportion. The presentation of the beads was: O, O, O, B, O, O, O, B, O, O, O, B, O, B, O, O, O, O. Task 2 is the same, but the probability is 60%:40% and the colours are purple and green. The presentation of the beads was as follows: P, G, G, P, P, G, P, P, P, G, P, P, P, P, G, G, P, G, G. Task 3 has the same probability, 60%:40%, but instead of beads, the jars contain negative and positive adjectives in this order: N, P, P, N, N, P, N, N, N, P, N, N, N, N, P, P, N, P, P. Task 3 has socially relevant content and a greater emotional charge than the abstract tasks^5^. JTC was considered to be taking a decision after extracting one or two beads^56^. We code the 60:40 task and 60:40 salient task as *difficult* because the probability of choosing the most likely jar is lower and the 85:15 task as *easy*, since the probability is higher^57^. JTC bias is obtained when participants make their decisions based on two or fewer beads/comments^5,58^.

### Assessment of social cognition

Considering the SC, we assess three domains of SC as follows: *Emotional Processing (EP)*: the component of EP measured was emotional recognition for which we used the Emotional Recognition Test^59,60^, that is composed of 20 photographs that express ten basic and ten complex emotions. *Theory of Mind (ToM)*: ToM was assessed using three histories of the Hinting Task^61,62^. The selection of the histories was made according the good psychometric properties of the Spanish version^62^. *Attributional style (AS)*: AS was assessed with the Internal, Personal, and Situational Attributions Questionnaire (IPSAQ)^63^. The IPSAQ has 32 items, which describe 16 positive and 16 negative social situations. For each item, the respondent must select a single most probable causal explanation for the situation described. Then the respondent is required to decide (one option only) as to whether the cause is internal (something to do with the respondent), personal (something to do with another person or persons), or situational (something to do with the circumstances, or random). Two cognitive bias scores are derived: externalising bias (EB) and personalisation bias (PB). A positive EB score indicates strong self-serving biases (blaming yourself less for negative events than positive ones). Personalising bias (PB) indicates the proportion of external attributions for negative events, which are personal and not situational. Likewise, six types of attributions are derived through the total sum of each of the positive and negative items, depending on whether the respondents focus the causes on themselves, others, or circumstances: the internal attribution for positive events, the internal attribution for negative events, the personal attribution for positive events, the personal attribution for negative events, the situational attribution for positive events and, the situational attribution of negative events. *Social perception (SP)* has not been evaluated because we have not found a validated tool in Spanish at the time of this study.

### Clinical assessment

The Positive and Negative Syndrome Scale (PANSS)^64,65^ was used for the assessment of psychotic symptoms. The PANSS is a semi-structured interview that assesses positive, negative, and general symptoms of people with psychosis in a total of 30 items. Delusional ideas were assessed with the Peters Delusions Inventory 21^66,67^. The PDI-21 is a self-report designed for assessing delusional ideas, which is composed of a total of 21 items with a dichotomous response format (yes/no). The total score is the sum of positive responses to each item, yielding a maximum score of 21 points. The higher the score, the greater the delusional ideas or proneness to paranoia. In addition, for each of the items, there are three subscales that measure level of conviction, preoccupation, and distress. On these three subscales, the scoring system is Likert-type with 5 categories (1–5)^68^.

The Beck Depression Inventory-II (BDI-II)^69–71^ is a 21-item questionnaire that assesses the severity of depressive symptoms. Higher scores reflect higher levels of symptom severity.

Premorbid estimated Intelligence Quotient (IQ) was calculated with vocabulary subtest of the Wechsler Adult Intelligence Scales (WAIS-III)^72^.

### Ethics committee

The project was evaluated by the research and ethics committee of each centre involved in the recruitment and evaluation of the participants: Servicio Andaluz of Jaén, Málaga and Motril (Granada), Hospital de la Santa Creu i Sant Pau (Barcelona), Hospital Clínico Universitario de Valencia, Centro de Higiene Mental de les Corts (Barcelona), Salut Mental Parc Taulí (Sabadell), Institut d´Assistència Sanitària Girona, and the coordinating centre Parc Sanitari Sant Joan de Déu (Sant Boi). The study participants were informed verbally and in writing of the objectives, content and duration of the evaluations. Then, the written informed consent was signed by all participants.

### Statistical analysis

We conducted statistical analyses in three stages. First, we obtained descriptive statistics and sociodemographic and clinical variables of the sample. Second, we performed a Student’s t-test to investigate the associations between people that did JTC and who did not, with three SC subdomains (ER, AS, ToM). The effect sizes of all comparisons were calculated with Cohen’s d using pooled SD. We did not perform multiple comparison corrections due to the exploratory nature of this study^73,74^. We performed a binary logistic regression analysis with the stepwise method for each of the three tasks of the JTC as the dependent variables, and the subdomains of the SC that were significant in the univariate analysis as independent variables, controlling for positive (PANSS) and depressive (BDI) symptoms and, estimated premorbid IQ (WAIS-III). We controlled for these variables, considering the previous findings about the relationship between positive symptoms and JTC^1,17,75^, JTC and IQ^18,19,43^, and SC (almost AS) and depressive symptoms^47^. All the analyses were performed using the SPSS v24 program^46^.

### Reporting summary

Further information on research design is available in the Nature Research Reporting Summary linked to this article.

## Supplementary information

Supp Info Description of the Spanish metacognition group

REPORTING SUMMARY

## Data Availability

The data that support the findings of this study are available from the corresponding author upon reasonable request.

## References

[1] Dudley, R. et al. ‘Jumping to conclusions’ in first-episode psychosis. *Early Intervention Psychiatry.* **5**, 50–56 (2011). 10.1111/j.1751-7893.2010.00258.x 21272275

[2] Garety, P. et al. Reasoning, emotions, and delusional conviction in psychosis. *J. Abnorm. Psychol.* **114**, 373–384 (2005). 10.1037/0021-843X.114.3.373 16117574

[3] Rodriguez V (2019). Jumping to conclusions at first onset of psychosis predicts longer admissions, more compulsory admissions and police involvement over the next 4 years: the GAP study. Psychol. Med..

[4] Dudley, R. et al. ‘Jumping to conclusions’ in first-episode psychosis: A longitudinal study. *Br. J. Clin. Psychol.* **52**, 380–393 (2013). 10.1111/bjc.12023 24117911

[5] Dudley R, Taylor P, Wickham S, Hutton P (2016). Psychosis, delusions and the ‘Jumping to Conclusions’ reasoning bias: A systematic review and meta-analysis. Schizophr. Bull..

[6] Winton-Brown TT (2015). Misattributing speech and jumping to conclusions: A longitudinal study in people at high risk of psychosis. Eur. Psychiatry.

[7] Falcone, M. A. et al. Jumping to Conclusions, Neuropsychological functioning, and delusional beliefs in first episode psychosis. *Schizophr. Bull.* (2015) 10.1093/schbul/sbu104. 10.1093/schbul/sbu104 PMC4332946 25053654

[8] Peters E, Garety P (2006). Cognitive functioning in delusions: A longitudinal analysis. Behav. Res. Ther..

[9] Garety PA, Freeman D (2013). The past and future of delusions research: From the inexplicable to the treatable. Br. J. Psychiatry.

[10] McLean BF, Mattiske JK, Balzan RP (2018). Towards a reliable repeated-measures beads task for assessing the jumping to conclusions bias. Psychiatry Res..

[11] Freeman, D., Pugh, K. & Garety, P. Jumping to conclusions and paranoid ideation in the general population. *Schizophr. Res.* **102**, 254–260 (2008). 10.1016/j.schres.2008.03.020 18442898

[12] Fine C, Gardner M, Craigie J, Gold I (2007). Hopping, skipping or jumping to conclusions? Clarifying the role of the JTC bias in delusions. Cognitive Neuropsychiatry.

[13] McLean, B. F., Mattiske, J. K. & Balzan, R. P. Association of the jumping to conclusions and evidence integration biases with delusions in psychosis: A detailed meta-analysis. *Schizophr. Bull.* **43**, 344–354 (2017). 10.1093/schbul/sbw056 PMC5605251 27169465

[14] Menon M, Mizrahi R, Kapur S (2008). ‘Jumping to conclusions’ and delusions in psychosis: Relationship and response to treatment. Schizophr. Res..

[15] Sanford, N., Lecomte, T., Leclerc, C., Wykes, T. & Woodward, T. S. Change in jumping to conclusions linked to change in delusions in early psychosis. *Schizophr. Res. 1(147), 207-208* (2013) 10.1016/j.schres.2013.02.042. 10.1016/j.schres.2013.02.042 23561297

[16] Ho-wai So, S., Peters, E. R., Swendsen, J., Garety, P. A. & Kapur, S. Changes in delusions in the early phase of antipsychotic treatment - An experience sampling study. *Psychiatry Res.* **215**, 568–573 (2014). 10.1016/j.psychres.2013.12.033 24412352

[17] Díaz-cutraro, L. et al. Jumping to conclusions is differently associated with specific subtypes of delusional experiences: An exploratory study in first-episode psychosis. *Schizophr. Res.* **228**, 357–359 (2021). 10.1016/j.schres.2020.12.037 33548835

[18] Ochoa, S. et al. Relation between jumping to conclusions and cognitive functioning in people with schizophrenia in contrast with healthy participants. *Schizophr. Res.* **159**, 211–217 (2014). 10.1016/j.schres.2014.07.026 25112159

[19] González LE (2018). Neuropsychological functioning and jumping to conclusions in recent onset psychosis patients. Schizophr. Res..

[20] Garety, P., Waller, H., Emsley, R., Jolley, S., Kuipers, E., Bebbington, P. & Freeman, D. Cognitive mechanisms of change in delusions: an experimental investigation targeting reasoning to effect change in paranoia. *Schizophr. Bull.* **41**, 400–410 (2015). 10.1093/schbul/sbu103 PMC4332945 25053650

[21] Tripoli G (2021). Jumping to conclusions, general intelligence, and psychosis liability: findings from the multi-centre EU-GEI case-control study. Psychol. Med..

[22] Healey KM, Bartholomeusz CF, Penn DL (2016). Deficits in social cognition in first episode psychosis: A review of the literature. Clin. Psychol. Rev..

[23] Adolphs R (1999). Social cognition and the human brain. Trends Cognitive Sci..

[24] Fett AKJ (2011). The relationship between neurocognition and social cognition with functional outcomes in schizophrenia: A meta-analysis. Neurosci. Biobehavioral Rev..

[25] Sergi MJ (2007). Social cognition in schizophrenia: Relationships with neurocognition and negative symptoms. Schizophr. Res..

[26] van Hooren S (2008). Social cognition and neurocognition as independent domains in psychosis. Schizophr. Res..

[27] Grossman MJ, Bowie CR (2020). Jumping to social conclusions?: The implications of early and uninformed social judgements in first episode psychosis. J. Abnorm. Psychol..

[28] Pinkham AE (2014). The social cognition psychometric evaluation study: Results of the expert survey and RAND Panel. Schizophr. Bull..

[29] Pinkham AE, Penn DL, Green MF, Harvey PD (2016). Social cognition psychometric evaluation: Results of the initial psychometric study. Schizophr. Bull..

[30] Pinkham AE, Harvey PD, Penn DL (2018). Social cognition psychometric evaluation: Results of the final validation study. Schizophr. Bull..

[31] Tripoli G (2015). Working memory, jumping to conclusions and emotion recognition: A Possible Link in First Episode Psychosis (Fep). Eur. Psychiatry..

[32] Tripoli, G. et al. S77. Jumping to conclusions and facial emotion recognition impairment in first-episode psychosis across europe. in *Sixth Biennial Conference Schizophrenia International Research Society (SIRS)*. S354–S355 (Schizophr. Bull, 2018). 10.1093/schbul/sby018.864.

[33] Takeda T (2018). Effect of cognitive function on jumping to conclusion in patients with schizophrenia. Schizophr. Res.: Cognition.

[34] Woodward TS, Mizrahi R, Menon M, Christensen BK (2009). Correspondences between theory of mind, jumping to conclusions, neuropsychological measures and the symptoms of schizophrenia. Psychiatry Res..

[35] Moritz S, Bentall RP, Kolbeck K, Roesch-Ely D (2018). Monocausal attribution and its relationship with reasoning biases in schizophrenia. Schizophr. Res..

[36] Rocha NBF, Queirós C (2013). Metacognitive and social cognition training (MSCT) in schizophrenia: A preliminary efficacy study. Schizophr. Res..

[37] Moritz S (2011). Further evidence for the efficacy of a metacognitive group training in schizophrenia. Behav. Res. Ther..

[38] Sauvé G, Lavigne KM, Pochiet G, Brodeur MB, Lepage M (2020). Efficacy of psychological interventions targeting cognitive biases in schizophrenia: A systematic review and meta-analysis. Clin. Psychol. Rev..

[39] Moritz S (2014). Sowing the seeds of doubt: A narrative review on metacognitive training in schizophrenia. Clin. Psychol. Revi..

[40] Ochoa S (2017). Randomized control trial to assess the efficacy of metacognitive training compared with a psycho-educational group in people with a recent-onset psychosis. Psychol. Med..

[41] Moritz S (2014). Sustained and ‘sleeper’ effects of group metacognitive training for schizophrenia a randomized clinical trial. JAMA Psychiatry.

[42] Merrin J, Kinderman P, Bentall RP (2007). ‘Jumping to conclusions’ and attributional style in persecutory delusions. Cognitive Ther. Res..

[43] Garety P (2013). Neuropsychological functioning and jumping to conclusions in delusions. Schizophr. Res..

[44] Moritz, S. et al. Different sides of the same coin? Intercorrelations of cognitive biases in schizophrenia. (2010) 10.1080/13546800903399993. 10.1080/13546800903399993 20146127

[45] Trower P, Chadwick P (1995). Pathways to defense of the self: A theory of two types of paranoia. Clin. Psychol.: Sci. Prac..

[46] IBM Corp. IBM SPSS Statistics for Windows, Version 24.0. (2016).

[47] Fornells-Ambrojo M, Garety PA (2009). Understanding attributional biases, emotions and self-esteem in ‘poor me’ paranoia: Findings from an early psychosis sample. Br. J. Clin. Psychol..

[48] Marley C, Jones J, Jones CA (2017). Testing the Trower and Chadwick model of paranoia: Is ‘poor-me’ and ‘bad-me’ paranoia acting as a defence?. Psychiatry Res..

[49] Garety PA (2017). SlowMo, a digital therapy targeting reasoning in paranoia, versus treatment as usual in the treatment of people who fear harm from others: Study protocol for a randomised controlled trial. Trials.

[50] Garety, P. et al. Effects of SlowMo, a blended digital therapy targeting reasoning, on paranoia among people with psychosis: A randomized clinical trial. *JAMA Psychiatry* (2021) 10.1001/jamapsychiatry.2021.0326. 10.1001/jamapsychiatry.2021.0326 PMC8027943 33825827

[51] García-mieres, H. & Jesús-romero, R. De. Beyond the cognitive insight paradox: Self-re flectivity moderates the relationship between depressive symptoms and general psychological distress in psychosis. In press. *Schizophr. Res.* (2020) 10.1016/j.schres.2020.05.027. 10.1016/j.schres.2020.05.027 32518005

[52] American Psychiatric Association (APA). *Diagnostic and Statistical Manual of Mental Disorders (DSM-IV-TR)*. (2000).

[53] Breitborde NJK, Srihari VH, Woods SW (2009). Review of the operational definition for first-episode psychosis. Early Intervention Psychiatry.

[54] Garety PA, Hemsley DR, Wessely S (1991). Reasoning in deluded schizophrenic and paranoid patients biases in performance on a probabilistic inference task. J Nervous Mental Dis..

[55] Dudley REJ, John CH, Young AW, Over DE (1997). The effect of self-referent material on the reasoning of people with delusions. Br. J. Clin. Psychol..

[56] Brett CMC (2007). Appraisals of Anomalous Experiences Interview (AANEX): a multidimensional measure of psychological responses to anomalies associated with psychosis. Br. J. Psychiatry Suppl..

[57] Ross RM, McKay R, Coltheart M, Langdon R (2015). Jumping to conclusions about the beads task? A meta-analysis of delusional ideation and data-gathering. Schizophr. Bull..

[58] Brett-Jones, J., Garety, P. & Hemsley, D. Measuring delusional experiences: A method and its application. *Br. J. Clin. Psychol.* (1987) 10.1111/j.2044-8260.1987.tb01359.x. 10.1111/j.2044-8260.1987.tb01359.x 3427248

[59] Huerta-Ramos, E. et al. Translation and validation of Baron Cohen’s Face Test in a general population from Spain. *Actas españolas de psiquiatría* in press (2020). 33969470

[60] Baron-Cohen S, Jolliffe T, Mortimore C, Robertson M (1997). Another advanced test of theory of mind: evidence from very high functioning adults with autism or Asperger Syndrome. J. Child Psychol. Psychiatry.

[61] Corcoran T, Goertz M (1995). Instructional capacity and high performance schools. Educational Researcher.

[62] Gil D, Fernández-Modamio M, Bengochea R, Arrieta M (2012). Adaptación al español de la prueba de teoría de la mente. Revista de Psiquiatria y Salud Mental.

[63] Kinderman P, Bentall RP (1996). A new measure of causal locus: The internal, personal and situational attributions questionnaire. Personality Individ. Diff..

[64] Kay SR, Fiszbein A, Opler LA (1987). The positive and negative Syndrome Scale for schizophrenia. Schizophr. Bull..

[65] Peralta V, Cuesta MJ (1994). Psychometric properties of the positive and negative syndrome scale (PANSS) in schizophrenia. Psychiatry Res..

[66] Peters ER, Joseph SA, Garety PA (1999). Measurement of delusional ideation in the normal population: Introducing the PDI (Peters et al. Delusions Inventory). Schizophr. Bull..

[67] Peters E, Joseph S, Day S, Garety P (2004). Measuring delusional ideation: The 21-item Peters et al. Delusions Inventory (PDI). Schizophr. Bull..

[68] Fonseca-Pedrero E, Paino M, Santarén-Rosell M, Lemos-Giráldez S, Muñiz J (2012). Psychometric properties of the Peters et al. Delusions Inventory 21 in college students. Compr. Psychiatry.

[69] Beck, A. T. and Steer, R. A. and Brown, G. K. *Beck depression inventory (BDI-II)*. (1996).

[70] Valero Rivas I (2018). Una Formulación Funcional Familiar Usando el Modelo Dinámico Maduracional del Apego y Adaptación. Revista de Psicoterapia.

[71] Sanz J, Perdigón AL, Vázquez C (2003). Adaptación española del Inventario para la Depresión de Beck-II (BDI-II): Propiedades psicométricas en población general. Clínica y salud.

[72] Wechsler, D. *WAIS-III: Escala de inteligencia de Wechsler para adultos-III. TEA*. (1999).

[73] Bender R, Lange S (2001). Adjusting for multiple testing - When and how?. J. Clin. Epidemiol..

[74] Nakagawa S (2004). A farewell to Bonferroni: The problems of low statistical power and publication bias. Behav. Ecol..

[75] So SHW, Kwok NTK (2015). Jumping to conclusions style along the continuum of delusions: Delusion-prone individuals are not hastier in decision making than healthy individuals. PLoS ONE.

[76] Endincott J (1976). The Global Assessment Scale: a procedure for measuring overall severity of psychiatric disturbance. Archives of General Psychiatry.

