# Supplementary material for: The relationship between jumping to conclusions and social cognition in first-episode psychosis
Source: Schizophrenia (Heidelb). 2022 Apr 20;8(1):39. doi: 10.1038/s41537-022-00221-3 (PMC9261088; doi:10.1038/s41537-022-00221-3)
Supplement: Supplementary file 1 — Supp Info Description of the Spanish metacognition group [file 41537_2022_221_MOESM1_ESM.docx]

**Supplementary Information File:**

Spanish Metacognition Study Group (SMSG): Acevedo A^1^, Alonso-Solís A^9^, Anglès J^6^, Ansó L^6^, Argany MA^6^, Aznar A^6^, Barajas A^6,17^, Barrigón ML^11^, Beltrán M^15^, Birulés, I^1^, Bogas JL^1^, Cabezas A^8^, Camprubí N^15^, Carbonero M^6^, Carrasco E^1^, Casañas R^6^, Cid J^15^, Conesa E^6^, Corripio I^9^, Cortes P^13^, de Apraiz A^1^, Delgado M^12^, Domínguez L^12^, Escartí MJ^7^, Escudero A^6^, Esteban Pinos I^12^, Ferrer-Quintero M^1,2,3,4^, Franco C^6^, Frigola-Capell E^15^ , Forns L^6^, García C^6^, Gonzalez-Casares R^12^, González-Higueras F^13^, González-Montoro ML^12^, González E^6^, Grasa-Bello E^9^, Guasp A^7^, Gutiérrez-Zotes^8^, Huerta-Ramos ME^1^, Huertas P^12^, Jiménez-Díaz A^13^, Lalucat LL^6^ , Legido T^10^, LLacer B^7^, López-Carrilero R^1,3^, López-Frutos A^7^, Lorente E^7^, Luengo A^7^, Mantecón N, Mas-Expósito L^6^, Montes M^14^, Montserrat C^10^, Moreno-Kustner B^14^, Moritz S^16^, Murgui E^6^, Nuñez M^1^, Ochoa S^1,3^, Palomer E^15^, Peláez T^1^, Planell K^15^, Planellas C^15^, Pleguezuelo-Garrote P^13^, Pousa E^9^, Renovell M^7^, Rubio R^6^, Ruiz-Delgado I^14^, Salas-Sender M^1^, San Emeterio M^6^, Sánchez E^6^, Sánchez-Alonso S^11^, Sanjuán J^7^, Sans B^6^, Sió H^6^, Teixidó M^6^, Torres P^13^, Vidiella M^6^, Vila MA^15^, Vila-Badia R^1^, Villegas F^6^.

1. Parc Sanitari Sant Joan de Déu, Sant Boi de Llobregat (Barcelona); 2. Departament de Psicologia Social i Psicologia Quantitativa. Universitat de Barcelona; 3. Investigación Biomédica en Red de Salud Mental (CIBERSAM); 4. Fundació Sant Joan de Déu, Esplugues de Llobregat (Barcelona); 5. Departament de Psicologia Clínica i de la Salut, Facultat de Psicologia, Universitat Autònoma de Barcelona, Bellaterra, Cerdanyola del Vallès, Spain. Serra Húnter fellow 6. Department of Research, Centre d'Higiene Mental Les Corts, Barcelona, Spain; 7. Psychiatry Service, Hospital Clínico Universitario de Valencia; 8. Hospital Universitari Institut Pere Mata, Institut d'Investigació Sanitària Pere Virgili (IISPV), Universitat Rovira i Virgili. Reus, Spain; 9. Department of Psychiatry, Hospital de la Santa Creu i Sant Pau, Institut d’Investigació Biomèdica-Sant Pau (IIB-Sant Pau), Universitat Autònoma de Barcelona; 10. Neuropsiquiatria i Addicions, Hospital del Mar. IMIM (Hospital del Mar Medical Research Institute). Barcelona; 11. Department of Psychiatry, IIS-Fundación Jiménez Díaz Hospital (Madrid); 12. Psychiatry Service, Area de Gestión Sanitaria Sur Granada, Motril (Granada) 13. Comunidad Terapéutica Jaén Servicio Andaluz de Salud; 14. Unidad de Salud Mental Comunitaria Malaga Norte; 15. Mental Health & Addiction Research Group. IdiBGi. Institut d’Assistencia Sanitària, Girona; 16. Department of Psychiatry and Psychotherapy, University Medical Center Hamburg,Hamburg (Germany); 17. Serra Húnter fellow. Department of Statistics and Operations Research, Polytechnic University of Catalonia.
