# Supplementary material for: The relationship between jumping to conclusions and social cognition in first-episode psychosis
Source: Schizophrenia (Heidelb). 2022 Apr 20;8(1):39. doi: 10.1038/s41537-022-00221-3 (PMC9261088; doi:10.1038/s41537-022-00221-3)
Supplement: Supplementary file 2 — REPORTING SUMMARY [file 41537_2022_221_MOESM2_ESM.pdf]

nature portfolio

Corresponding author(s): NPJSCHZ-00711R

Last updated by author(s): Sep 24, 2021

Reporting Summary

Nature Portfolio wishes to improve the reproducibility of the work that we publish. This form provides structure for consistency and transparency in reporting. For further information on Nature Portfolio policies, see our Editorial Policies and the Editorial Policy Checklist.

Statistics

For all statistical analyses, confirm that the following items are present in the figure legend, table legend, main text, or Methods section.

n/a	Confirmed	
☐	☒	The exact sample size (n) for each experimental group/condition, given as a discrete number and unit of measurement
☐	☒	A statement on whether measurements were taken from distinct samples or whether the same sample was measured repeatedly
☐	☒	The statistical test(s) used AND whether they are one- or two-sided<br>*Only common tests should be described solely by name; describe more complex techniques in the Methods section.*
☐	☒	A description of all covariates tested
☐	☒	A description of any assumptions or corrections, such as tests of normality and adjustment for multiple comparisons
☐	☒	A full description of the statistical parameters including central tendency (e.g. means) or other basic estimates (e.g. regression coefficient) AND variation (e.g. standard deviation) or associated estimates of uncertainty (e.g. confidence intervals)
☒	☐	For null hypothesis testing, the test statistic (e.g. F, t, r) with confidence intervals, effect sizes, degrees of freedom and P value noted<br>*Give P values as exact values whenever suitable.*
☒	☐	For Bayesian analysis, information on the choice of priors and Markov chain Monte Carlo settings
☒	☐	For hierarchical and complex designs, identification of the appropriate level for tests and full reporting of outcomes
☐	☒	Estimates of effect sizes (e.g. Cohen's d, Pearson's r), indicating how they were calculated

Our web collection on statistics for biologists contains articles on many of the points above.

Software and code

Policy information about availability of computer code

Data collection	Data collection was performed using a paper data collection form. No computer software was used in this process.
Data analysis	All the statistical analyses were performed using the SPSS v24 program.

For manuscripts utilizing custom algorithms or software that are central to the research but not yet described in published literature, software must be made available to editors and reviewers. We strongly encourage code deposition in a community repository (e.g. GitHub). See the Nature Portfolio guidelines for submitting code & software for further information.

Data

Policy information about availability of data

All manuscripts must include a data availability statement. This statement should provide the following information, where applicable:

- Accession codes, unique identifiers, or web links for publicly available datasets
- A description of any restrictions on data availability
- For clinical datasets or third party data, please ensure that the statement adheres to our policy

The data that support the findings of this study are available from the corresponding author upon reasonable request.

March 2021

Field-specific reporting

Please select the one below that is the best fit for your research. If you are not sure, read the appropriate sections before making your selection.

☐ Life sciences ☒ Behavioural & social sciences ☐ Ecological, evolutionary & environmental sciences

For a reference copy of the document with all sections, see nature.com/documents/nr-reporting-summary-flat.pdf

Behavioural & social sciences study design

All studies must disclose on these points even when the disclosure is negative.

Study description	A cross-sectional study was performed based on baseline data from a large multicentre clinical trial (Ochoa, 2017). The main study was recorded in Clinical Trials (Identifier: NCT02340559).
Research sample	The sample was composed of 121 patients with a first-episode psychosis recruited at one of the nine participating mental health centres: Servicio Andaluz de Jaén, Málaga y Motril (Granada), Hospital de la Santa Creu i Sant Pau (Barcelona), Hospital Clínico Universitario de Valencia, Centro de Higiene Mental de les Corts (Barcelona), Salut Mental Parc Taulí (Sabadell), Institut d´Assistència Sanitària Girona, and the coordinating centre Parc Sanitari Sant Joan de Déu (Sant Boi).
Sampling strategy	For the present study, the baseline sample from the main research (Ochoa, 2017) was taken. The sample of people with first-episode psychosis was selected following these criteria:<br>Regarding the proposal of Breitborde (2009), inclusion criteria were: (1) a diagnosis of schizophrenia, psychotic disorder not otherwise specified, delusional disorder, schizoaffective disorder, brief psychotic disorder, or schizophreniform disorder (according to DSM-IV-TR); (2) <5 years from the onset of symptoms; (3) PANSS scores in delusions, grandiosity, or suspicions of ≥4 during the previous year; and (4) age between 17 and 45 years. Exclusion criteria were: (1) a traumatic brain injury, dementia, or intellectual disability (premorbid IQ ≤70; (2) substance dependence; and (3) PANSS scores in hostile and uncooperative of ≥ 5 and in suspiciousness ≥ 6.
Data collection	The data collection procedure was carried out by psychologists trained in assessment. A sociodemographic questionnaire was included that collected relevant information on the description of the samples. A battery of questionnaires relating to JTC, SC and clinical measures was included. All data collected was done with pencil and paper, and through face-to-face interviews in each of the participating centers.
Timing	2012-2014
Data exclusions	In this study we have taken the total number of patients who completed the Jumping to conclusions task (Beads task) at baseline of the main study (Ochoa, 2017). Only one case that had not completed the Beads Task was excluded. The total baseline sample of the main study is n=122, and the total sample of the current study is n=121.
Non-participation	This study is cross-sectional and is based on a previous baseline study. For this reason, this item is not applicable.
Randomization	This study is cross-sectional and is based on a previous baseline study. For this reason, this item is not applicable.

Reporting for specific materials, systems and methods

We require information from authors about some types of materials, experimental systems and methods used in many studies. Here, indicate whether each material, system or method listed is relevant to your study. If you are not sure if a list item applies to your research, read the appropriate section before selecting a response.

Materials & experimental systems

n/a	Involved in the study	
☒	☐	Antibodies
☒	☐	Eukaryotic cell lines
☒	☐	Palaeontology and archaeology
☒	☐	Animals and other organisms
☒	☐	Human research participants
☒	☐	Clinical data
☒	☐	Dual use research of concern

Methods

n/a	Involved in the study	
☒	☐	ChIP-seq
☒	☐	Flow cytometry
☒	☐	MRI-based neuroimaging
